# Effect of Joule Heating and Thermal Radiation in Flow of Third Grade Fluid over Radiative Surface

**DOI:** 10.1371/journal.pone.0083153

**Published:** 2014-01-15

**Authors:** Tasawar Hayat, Anum Shafiq, Ahmed Alsaedi

**Affiliations:** 1 Department of Mathematics, Quaid-i-Azam University, Islamabad, Pakistan; 2 Nonlinear Analysis and Applied Mathematics (NAAM) Research Group, Faculty of Science, King Abdulaziz University, Jeddah, Saudi Arabia; Plymouth University, United Kingdom

## Abstract

This article addresses the boundary layer flow and heat transfer in third grade fluid over an unsteady permeable stretching sheet. The transverse magnetic and electric fields in the momentum equations are considered. Thermal boundary layer equation includes both viscous and Ohmic dissipations. The related nonlinear partial differential system is reduced first into ordinary differential system and then solved for the series solutions. The dependence of velocity and temperature profiles on the various parameters are shown and discussed by sketching graphs. Expressions of skin friction coefficient and local Nusselt number are calculated and analyzed. Numerical values of skin friction coefficient and Nusselt number are tabulated and examined. It is observed that both velocity and temperature increases in presence of electric field. Further the temperature is increased due to the radiation parameter. Thermal boundary layer thickness increases by increasing Eckert number.

## Introduction

There is a substantial interest of the recent researchers in the flows of non-Newtonian fluids. Such motivation in these fluids is mainly because of their use in the industrial and technological applications. Many materials like mud, pasta, personal care products, ice cream, paints, oils, cheese, asphalt etc. are non-Newtonian fluids. Most biological fluids with higher molecular weight components are also non-Newtonian in nature. The usual properties of polymer melts and solutions together with the desirable attributes of many polymeric solids, have given rise to the world-wide industry of polymer processing. The non-Newtonian fluids in particular have key importance in geophysics, chemical and nuclear industries, material processing, oil reservoir engineering, bioengineering and many others. Rheological properties of all the non-Newtonian fluids cannot be predicted using single constitutive equation (unlike the case of viscous fluids). Therefore many models of non-Newtonian fluids are based either on “natural” modifications of established macroscopic theories or molecular considerations. The additional rheological parameter in the constitutive equations of non-Newtonian fluids are the main culprit for the lack of analytical solutions. The resulting equations are more complex and higher order than the Navier-Stokes equations. Hence these equations have been attracted from modelling as well as solutions point of view. The advancement in the study of non-Newtonian fluids has been made even by the recent investigators (See [1]–[10] and many studies therein).

The flow induced over a stretching surface is very well documented problem in fluid mechanics. It is encountered in extrusion of polymer sheet from a die, glass fiber and paper production, continuous casting, cooling of metallic plate in a bath etc. Such flow problem in presence of heat transfer has been attracted by the researchers due to its applications in polymer processing technology. The quality of end product in industry depends upon both the stretching and cooling rates. Further, the thermal radiation effect has pivotal role in nuclear plants, gas turbines and devices for satellites, space vehicles, aircraft etc. The literature on this topic is quite sizeable. Rana and Bhargava [11] presented the numerical analysis for heat transfer of nanofluid over a nonlinearly stretching sheet. Bhattacharyya et al. [12] analyzed the solutions of boundary layer flow of viscoelastic fluid and heat transfer over a stretching sheet with internal heat generation or absorption. Makinde and Aziz [13] numerically studied the boundary layer flow of viscous nanofluid bounded by a stretching sheet. They considered the transport equation which includes the effects of Brownian motion and thermophoresis. Mandal and Mukhopadhyay [14] considered the boundary layer flow and heat transfer towards an exponentially stretching porous sheet embedded in a porous medium with variable surface heat flux. They found that the momentum and thermal boundary layer thickness decrease with increasing exponential parameter. Hayat et al. [15] examined the heat transfer in flow of second grade fluid over a stretching sheet. Thermal radiation effect in the boundary layer flow by stretching surface has been explored by Sajid and Hayat [16]. Bhattacharyya [17] discussed the unsteady stagnation point flow towards a stretching surface. Effect of heat transfer in flow over an exponentially stretching surface has been explored by Mukhopadhyay [18]. The radiation effect in flow of micropolar fluid towards a stretching surface is addressed by Hussain et al. [19]. Rashidi et al. [20] developed approximate solutions for heat transfer analysis in flow of micropolar fluid. Moreover, the interest in the study of magnetohydrodynamic flow for an electrically conducting fluid over heated surface is motivated by its great value in a wide range of engineering problems such as plasma studies, petroleum industries, MHD power generators, cooling of nuclear reactors, the boundary layer control in aerodynamics and crystal growth. Hence Turkyilmazoglu [21] found exact solution for magnetohydrodynamic flow of viscous fluid due to a rotating disk. Hayat and Nawaz [22] has investigated the Soret and Dufour effects in mixed convection three dimensional boundary flow of an electrically conducting second grade fluid over a vertical stretching sheet. Ahmad and Nazar [23] considered the problem of unsteady magnetohydrodynamic viscoelastic fluid flowing towards a stagnation point on a vertical surface. Pal and Mondal [24] discussed the hydromagnetic flow of viscous fluid over a stretching surface in presence of both electric and magnetic fields. Abel et al. [25] presented MHD flow analysis for viscoelastic fluid. Both viscous and Ohmic dissipations are presented in this attempt. More, the analysis here is made when magnetic and electric fields are present. Hayat and Qasim [26] considered radiation effect in MHD flow of second grade fluid over unsteady porous stretching surface. The effect of internal heat generation in hydromagnetic non-Darcy flow and heat transfer over a stretching surface with thermal radiation and Ohmic dissipation is examined by Olanrewaju [27]. Elbashbeshy et al. [28] numerically analyzed the problem of unsteady laminar two-dimensional MHD boundary layer flow and heat transfer of an incompressible viscous fluid over a porous surface in the presence of thermal radiation and internal heat generation or absorption. MHD flow caused by a rotating disk is presented by Rashidi et al. [29]. The well-known Jeffery-Hamel problem in presence of magnetic field is examined by Motsa et al. [30]. Most of the studies on MHD flow over a stretching surface with heat transfer do not take into account the effect of electric field and Ohmic dissipation. Very little exists yet about such aspects in the stretched flows of viscous fluids. Such consideration further narrowed down when non-Newtonian fluids have been considered. To our knowledge there is only one such attempt for viscoelastic fluid [25]. The fluid employed although exhibits the normal stress effects but it cannot describe the features of shear thinning or shear thickening. Having such in view, the flow of third grade fluid is considered. This fluid even can capture shear thinning/shear thickening effects for one-dimensional flow over a rigid surface. The main objective here is to analyze the two-dimensional flow of third grade fluid over an unsteady stretching sheet. The effects of both electric and magnetic fields are retained in the momentum and energy equations. Thermal radiation and Ohmic dissipation are taken into account. The solutions for velocity component and temperature are developed by homotopy analysis method (HAM) [31]–[40]. The plots of physical quantities of interest reflecting the novel features of embedded parameters in the problems are given and analyzed. Tables for skin friction coefficient and local Nusselt number are made and explained carefully.

## Mathematical Formulation

We examine the two-dimensional boundary layer flow of magnetohydrodynamic (MHD) third grade fluid over a porous stretching surface. Here the fluid is electrically conducting in the presence of applied magnetic 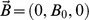 and electric 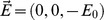 fields. The flow is because of stretching of sheet from a slit through two equal and opposite forces. The sheet velocity is taken linear parallel to the flow direction. The electric and magnetic fields obey the Ohm's law 
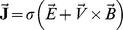
. Here 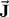 is the Joule current, 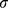 is the electrical conductivity and 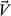 is the fluid velocity. The induced magnetic field and Hall current effects are ignored subject to small magnetic Reynolds number. Both the electric and magnetic fields contribute into the momentum and thermal boundary layer equations. The relevant equations in the aforestated conditions can be expressed as follows:
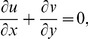
(1)

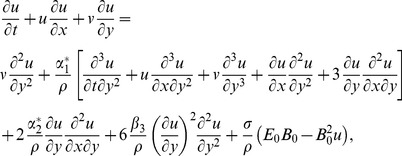
(2)

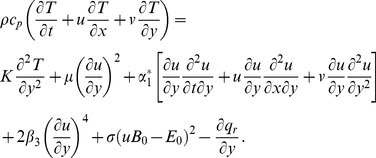
(3)In above equations 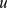 and 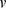 denote the velocity components in the 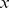 and 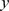 directions, 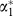, 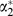 and 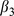 are the fluid parameters, 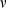 is the kinematic viscosity, 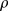 is the density of fluid, 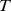 is the fluid temperature, 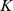 is the thermal conductivity of fluid, 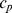 is the specific heat at constant pressure and the radiative heat flux 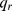 is first given by Sparrow and Cess [41] and Raptis [42]

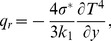
(4)where 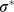 is the Stefan-Boltzmann constant and 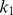 is the mean absorption coefficient. Through expansion of 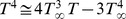, Eq. (3) becomes
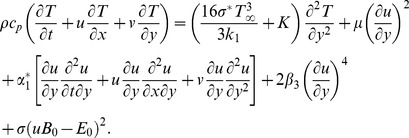
(5)The subjected conditions can be mentioned as follows:
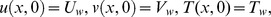


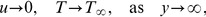
(6)with 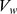 defined by
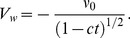
(7)Here the mass transfer at surface with 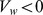 is for injection and 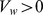 for suction. Also the stretching velocity 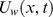 and the surface temperature 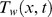 are taken in the forms:
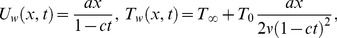
(8)where 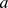 and 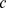 are the constants with 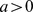 and 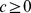 (i.e 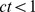).

If 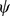 is the stream function then defining
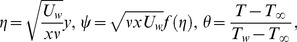
(9)

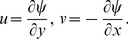
(10)The incompressibility condition is identically satisfied and the resulting problems for 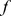 and 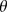 are reduced into the following forms
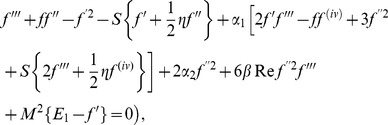
(11)

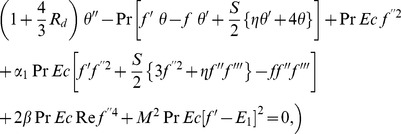
(12)

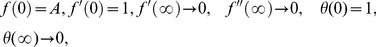
(13)with
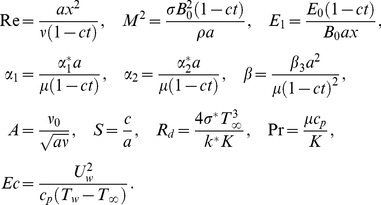
(14)Here 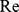 denotes the Reynolds number, 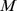 the magnetic parameter, 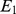 is the electric parameter, 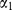 and 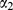 and 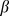 are the fluid parameters, 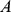 is the suction parameter, 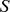 is the unsteadiness parameter, 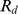 is the radiation parameter, 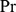 is the Prandtl number and 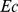 is the Eckert number.

The local skin friction coefficient is defined as
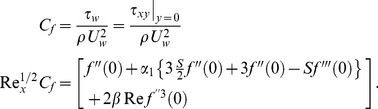
(15)The Nusselt number is given by
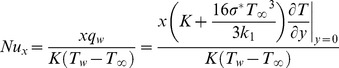


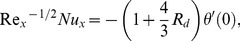
(16)in which 
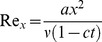
 is the local Reynolds number.

## Solutions

The velocity and temperature can be expressed in the set of base functions
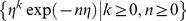
(17)can be expressed as follows
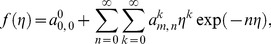
(18)

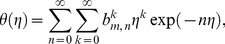
(19)where 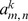 and 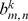 are the coefficients.

The initial guesses 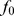 and 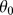 in homotopy solutions are taken through the expressions
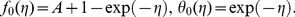
(20)The auxiliary linear operators and their associated properties are
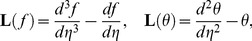
(21)satisfy the following properties
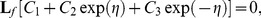
(22)

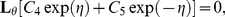
(23)Where 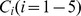 depict the arbitrary constants.

The zeroth order problems are
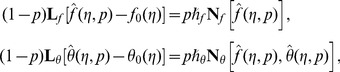
(24)

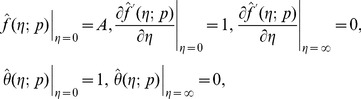
(25)with non-linear operators 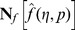 and 
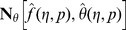
 defined by
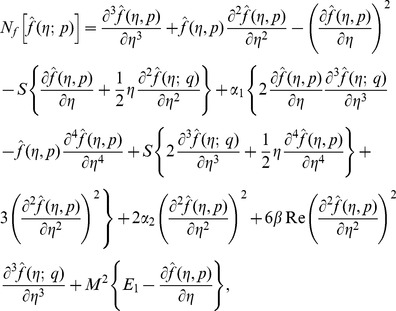
(26)

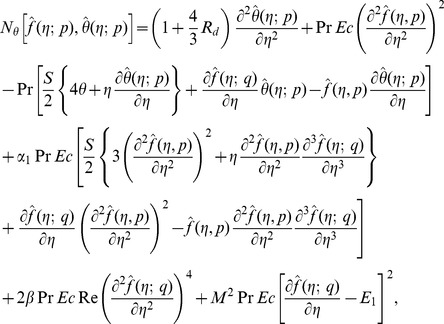
(27)in which 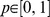 indicates the embedding parameter and 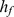 and 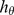 the nonzero auxiliary parameters.

Setting 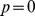 and 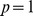 we have
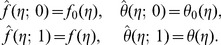
(28)When 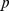 increases from 0 to 1, 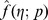 and 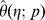 deforms from the initial solutions 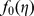 and 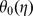 to the final solutions 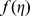 and 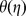, respectively. Taylor series, of 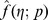 and 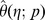 gives
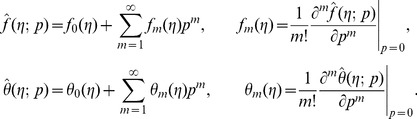
(29)The auxiliary parameters are properly chosen such that the series solutions converge at 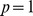. Therefore
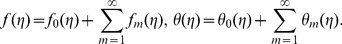
(30)The 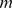
th-order deformation problems are
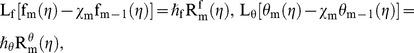
(31)

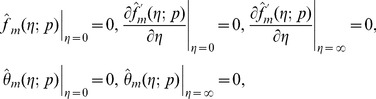
(32)

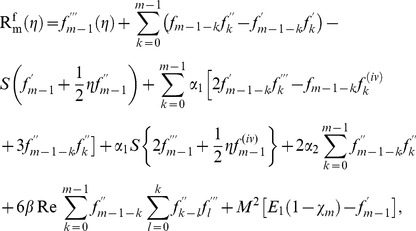
(33)

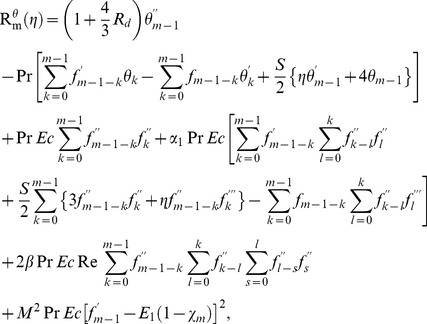
(34)

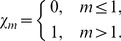
(35)The general solutions of the Eqs. (31)–(32) are
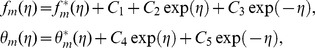
in which 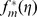 and 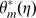 denote the special solutions.

## Convergence of the Derived Solutions

**Figure 1:**
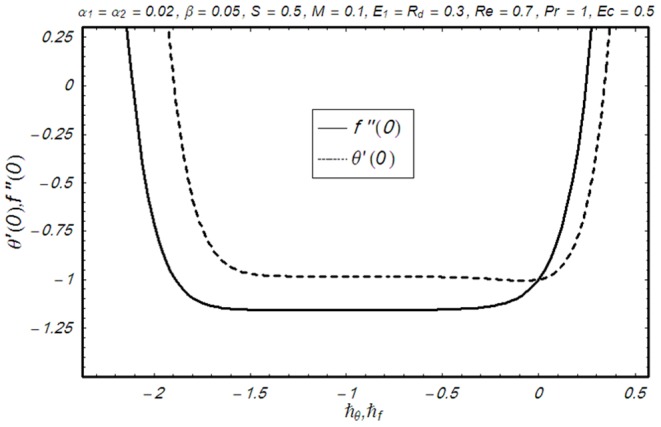
ħ-curves of the functions f″(0) and θ′(0) at 10^th^ order of approximation.

We note that the series solutions (33) and (34) contain the non-zero auxiliary parameters 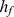 and 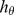. These parameters are useful in adjusting and controlling the convergence. The 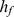 and 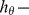
curves are plotted for 10^th^ order of approximation in Fig. 1 for the suitable ranges of the auxiliary parameters. Here the suitable values for 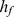 and 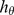 are 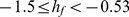, 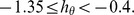 Furthermore, convergence of series solution is checked and shown in Table 1. Note that the series solutions converge at 26th order of approximation up to 6 decimal places.

**Table 1:** Convergence of homotopy solutions when 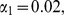 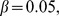 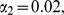 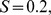 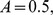 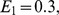 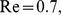 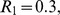 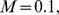 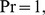 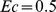.

Order of approximation	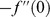	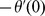
1	1.0419	1.0059
2	1.0720	1.0079
5	1.1210	1.0041
10	1.1442	0.99450
12	1.1458	0.99211
14	1.1458	0.99051
40	1.1458	0.99051

## Results and Discussion

**Figure 2:**
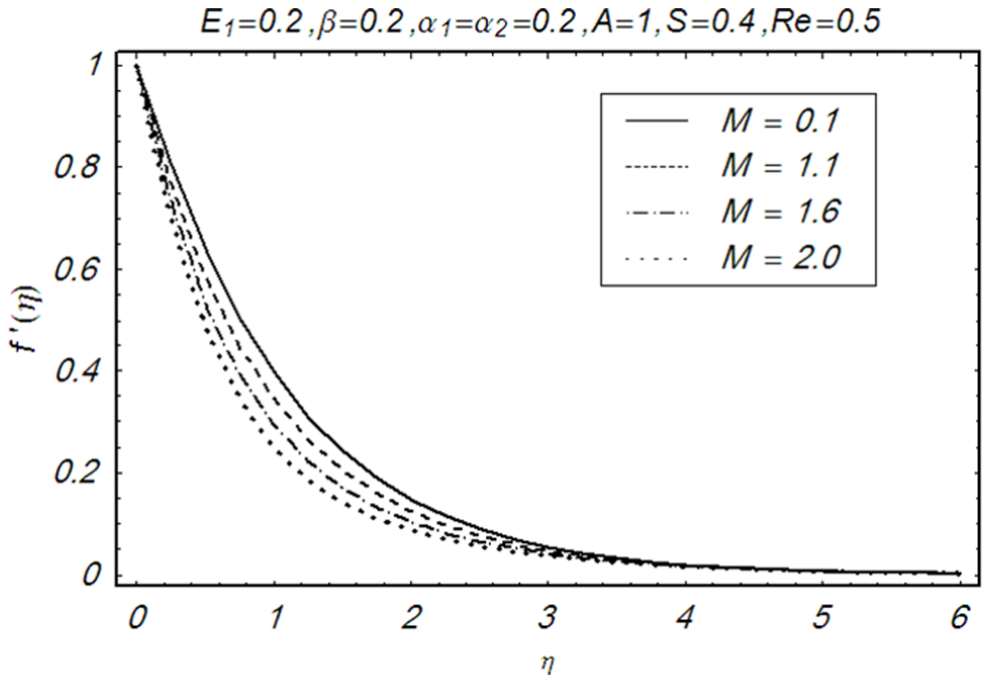
Influence of M on f′(η).

**Figure 3:**
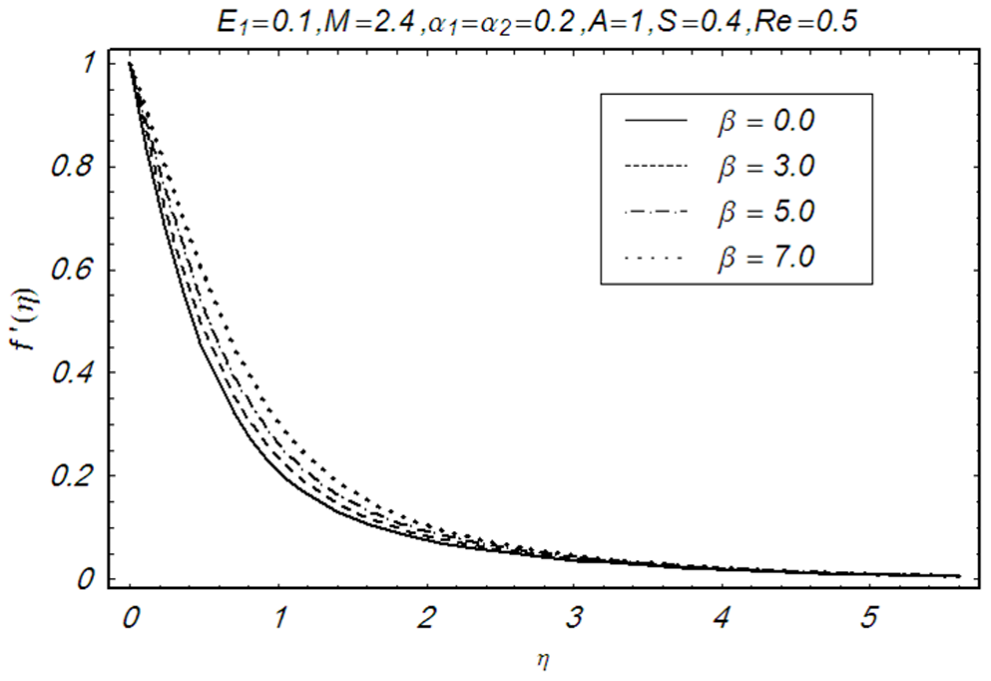
Influence of β on f′(η).

**Figure 4:**
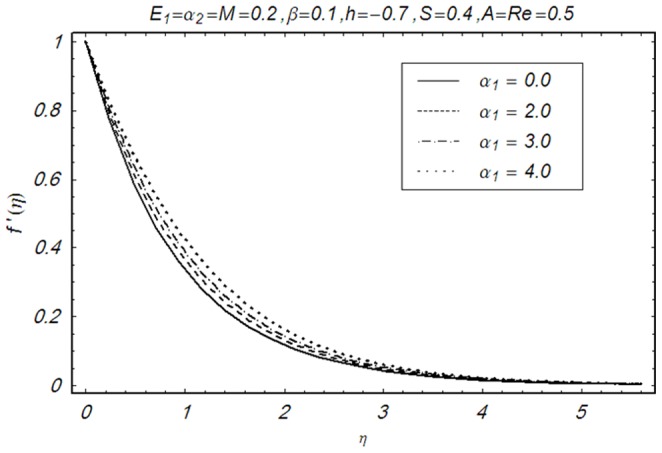
Influence of α_1_ on f′(η).

**Figure 5:**
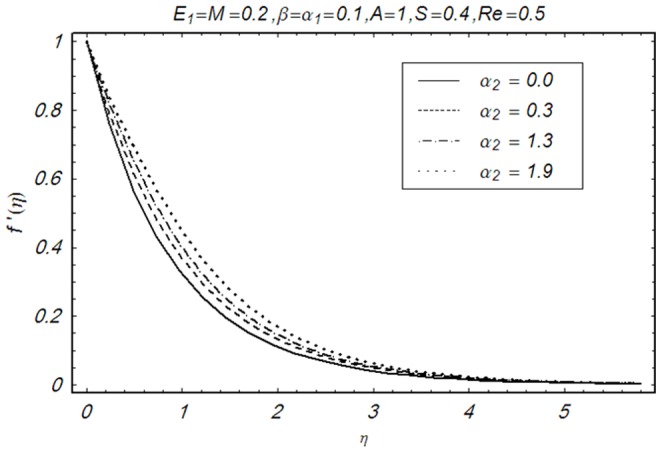
Influence of α_2_ on f′(η).

**Figure 6:**
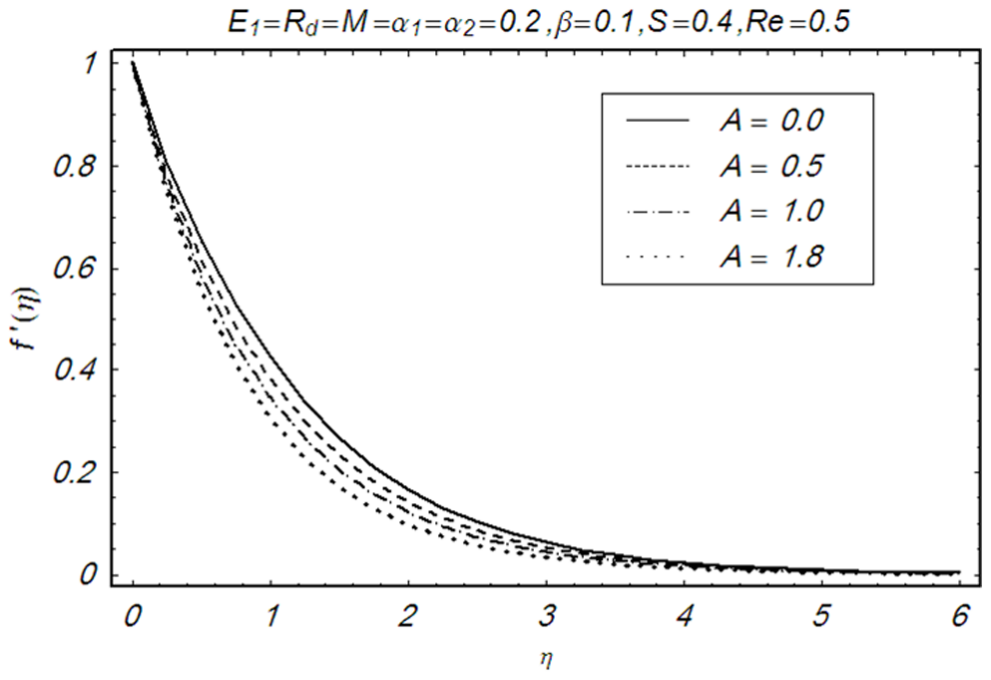
Influence of A on f′(η).

**Figure 7:**
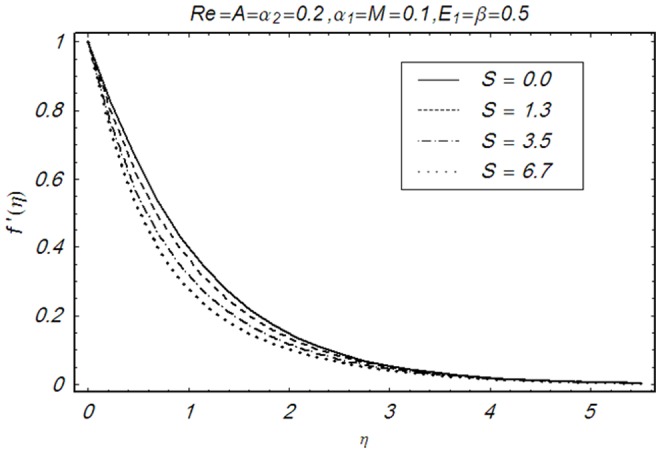
Influence of S on f′(η).

**Figure 8:**
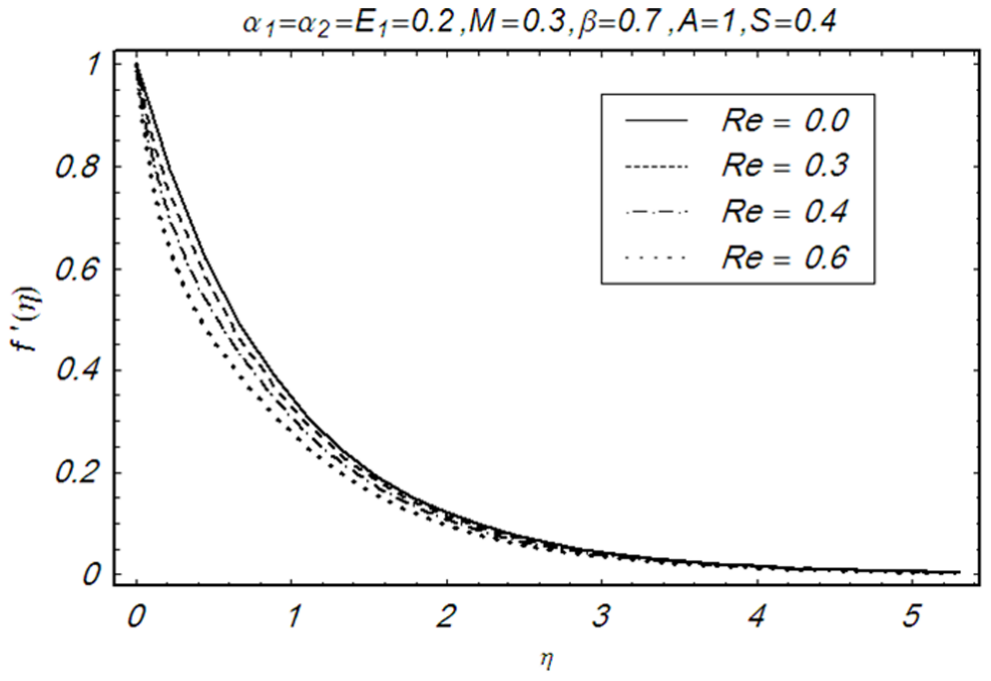
Influence of Re on f′(η).

**Figure 9:**
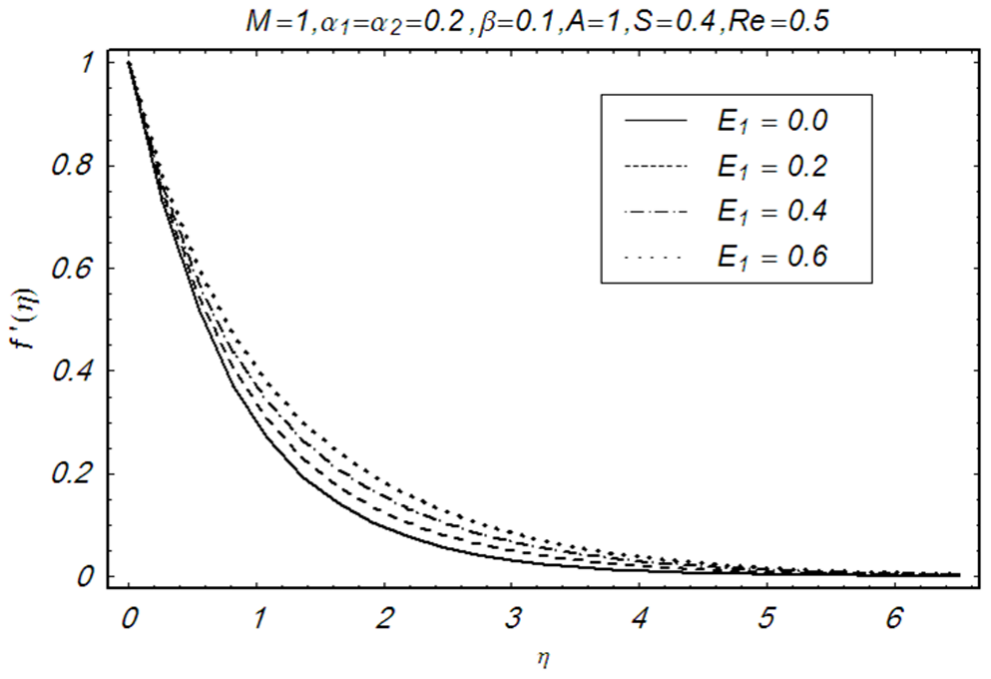
Influence of E_1_ on f′(η).

**Figure 10:**
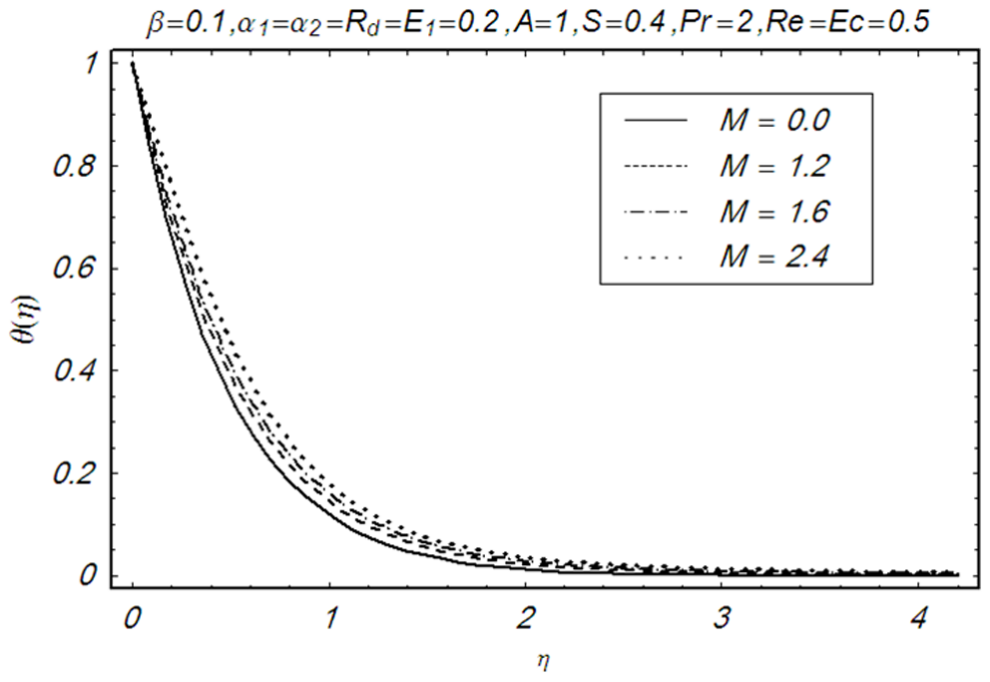
Influence of M on θ(η).

**Figure 11:**
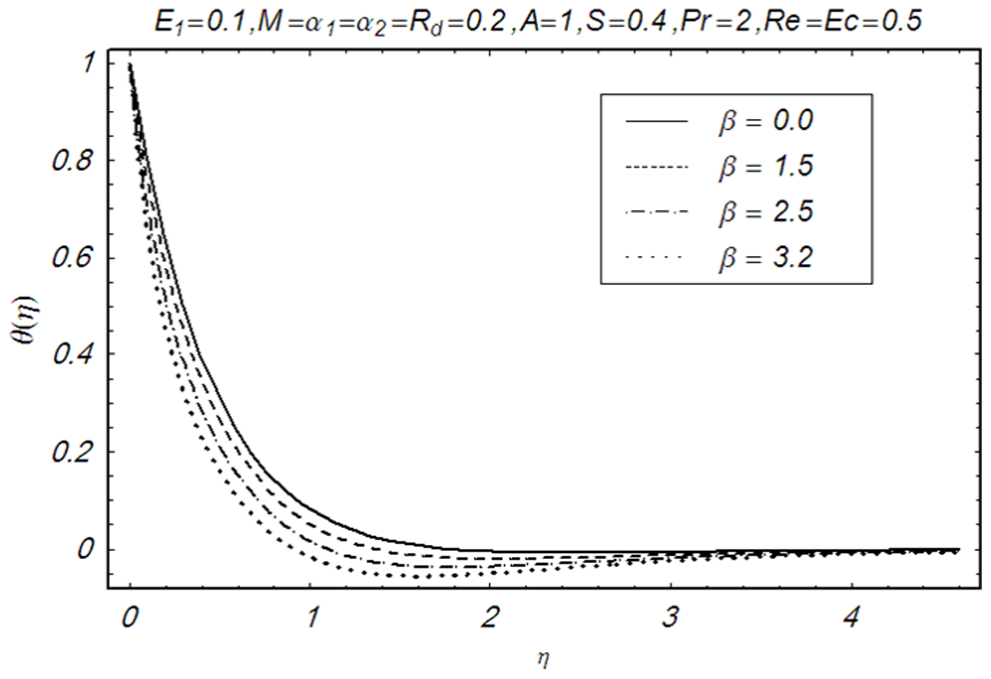
Influence of β on θ(η).

**Figure 12:**
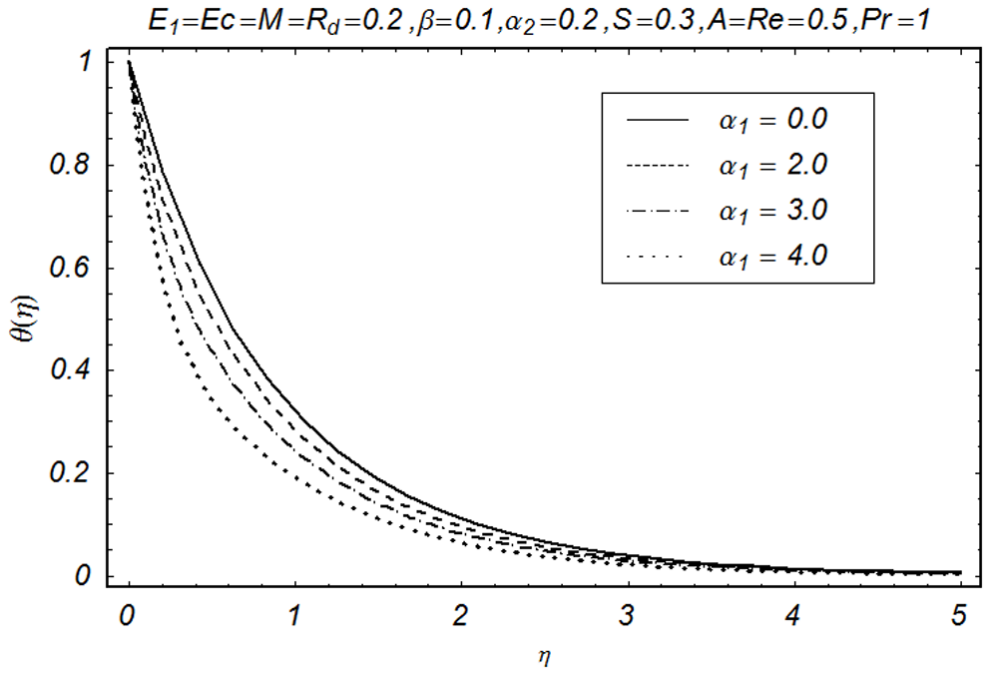
Influence of α_1_ on θ(η).

**Figure 13:**
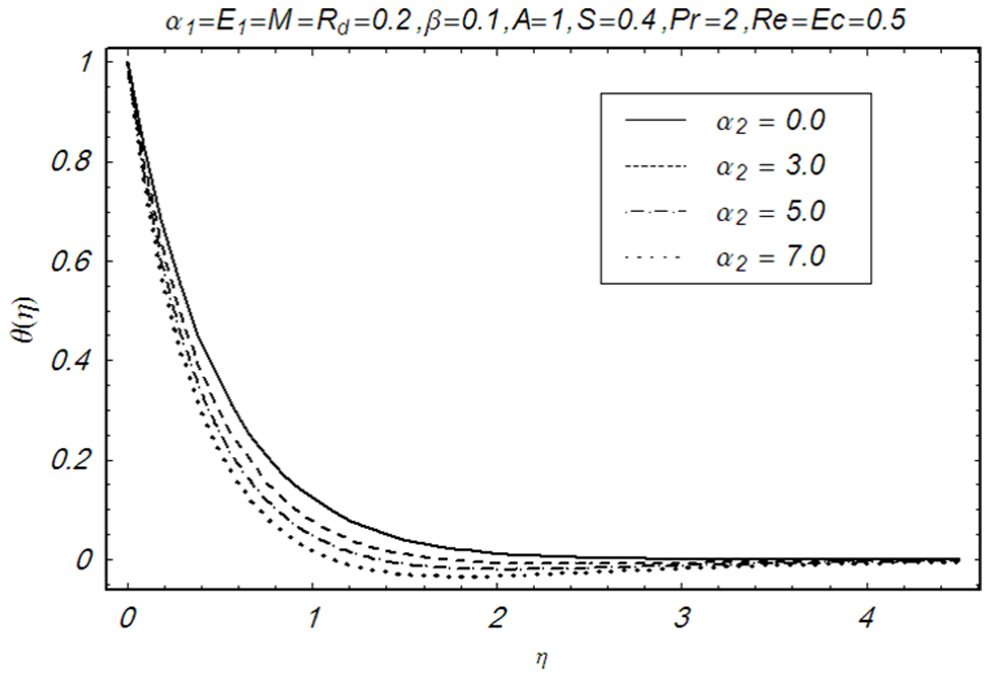
Influence of α_2_ on θ(η).

**Figure 14:**
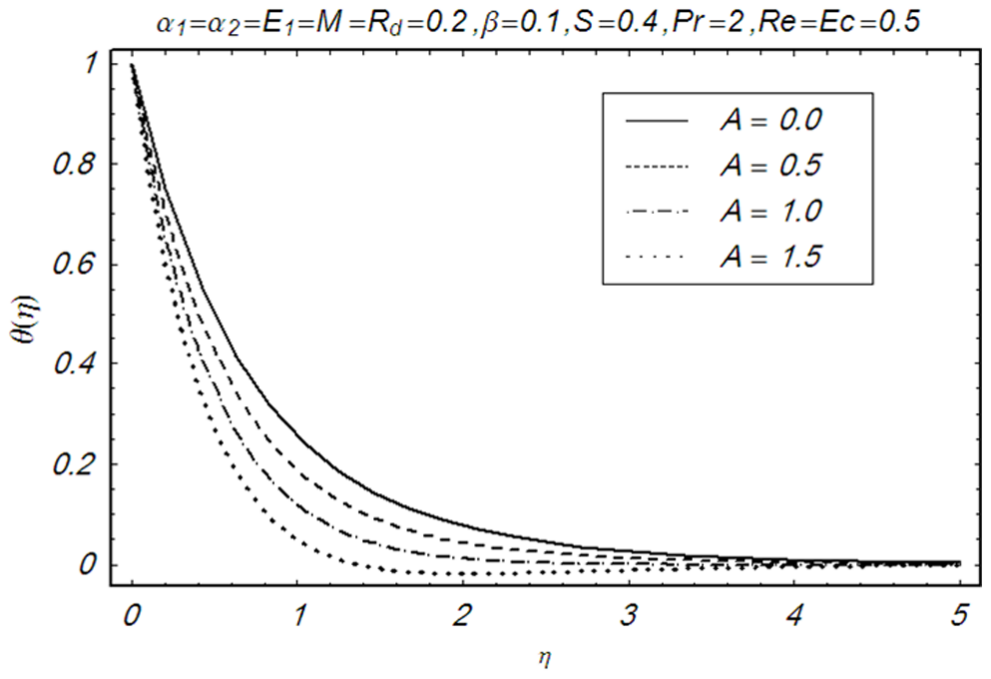
Influence of A on θ(η).

**Figure 15:**
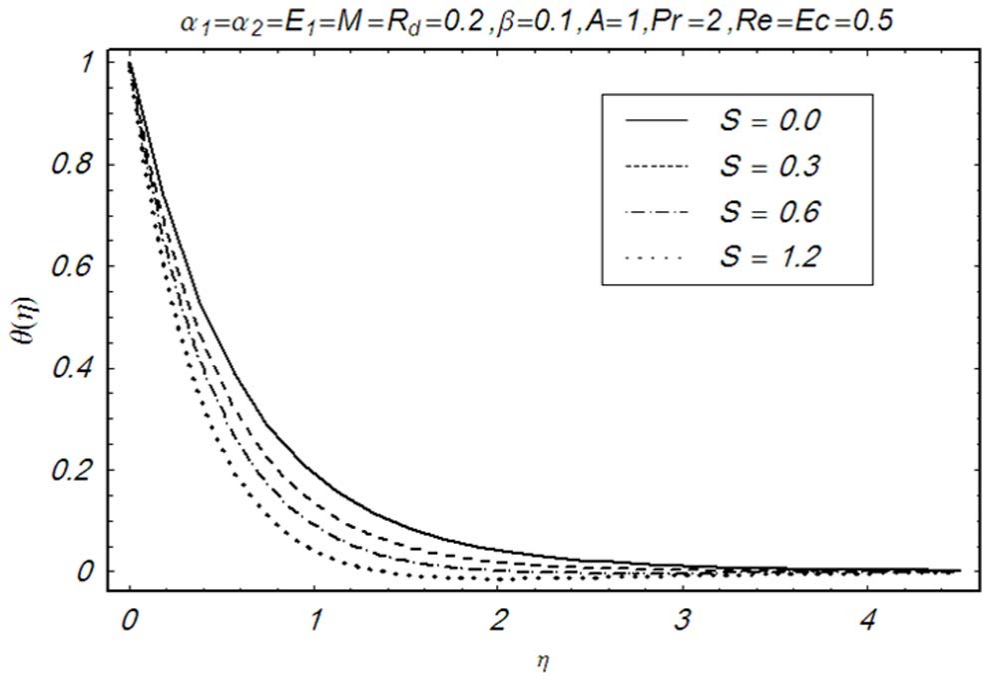
Influence of S on θ(η).

**Figure 16:**
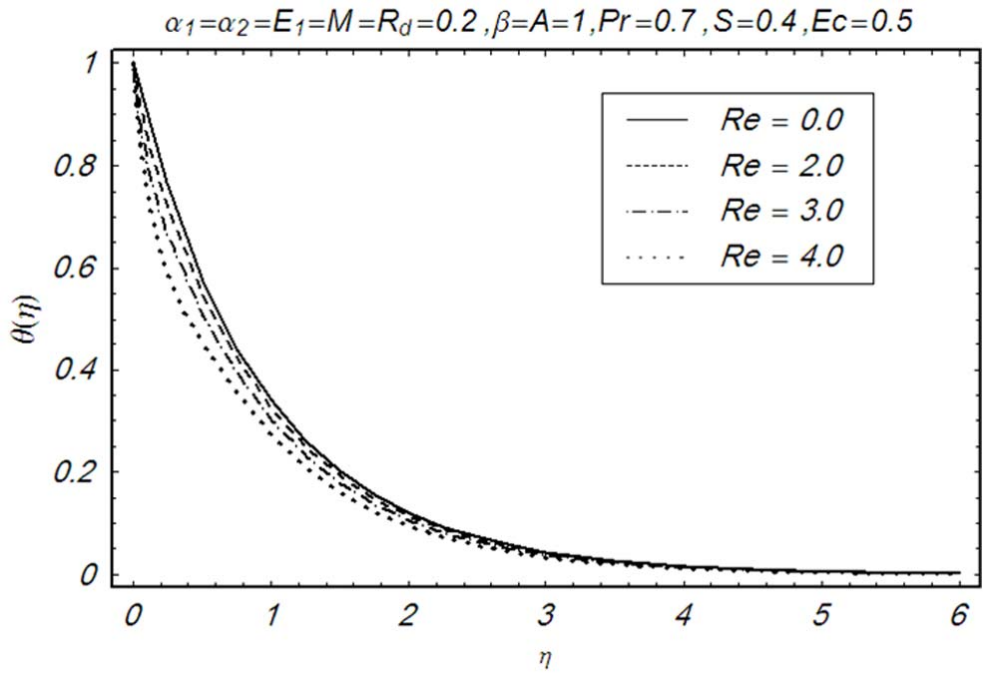
Influence of Re on θ(η).

**Figure 17:**
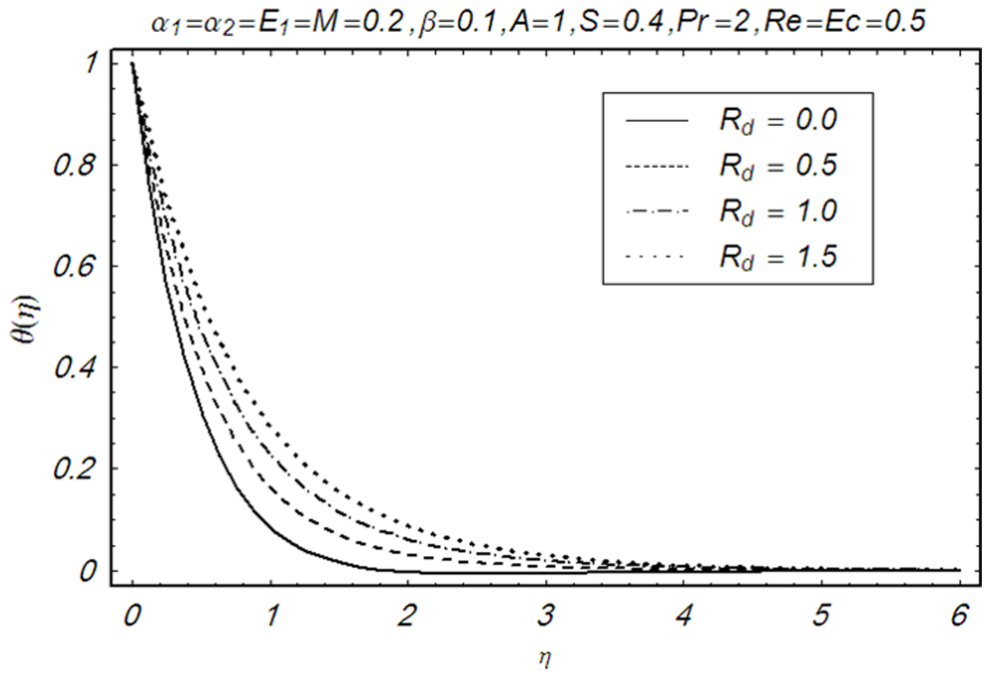
Influence of R_d_ on θ(η).

**Figure 18:**
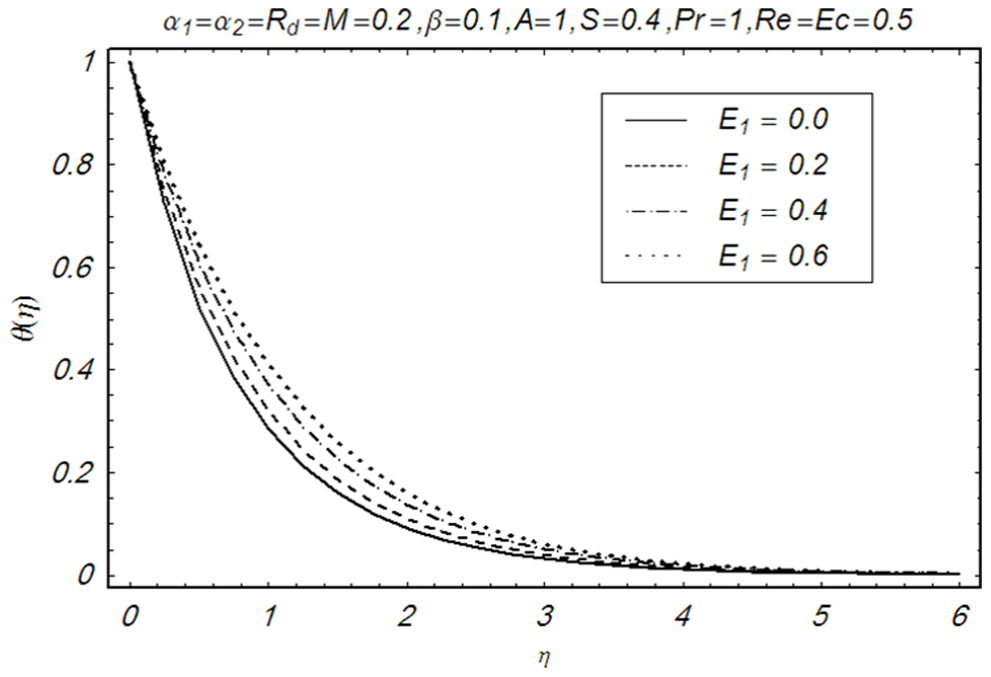
Influence of E_1_ on θ(η).

**Figure 19:**
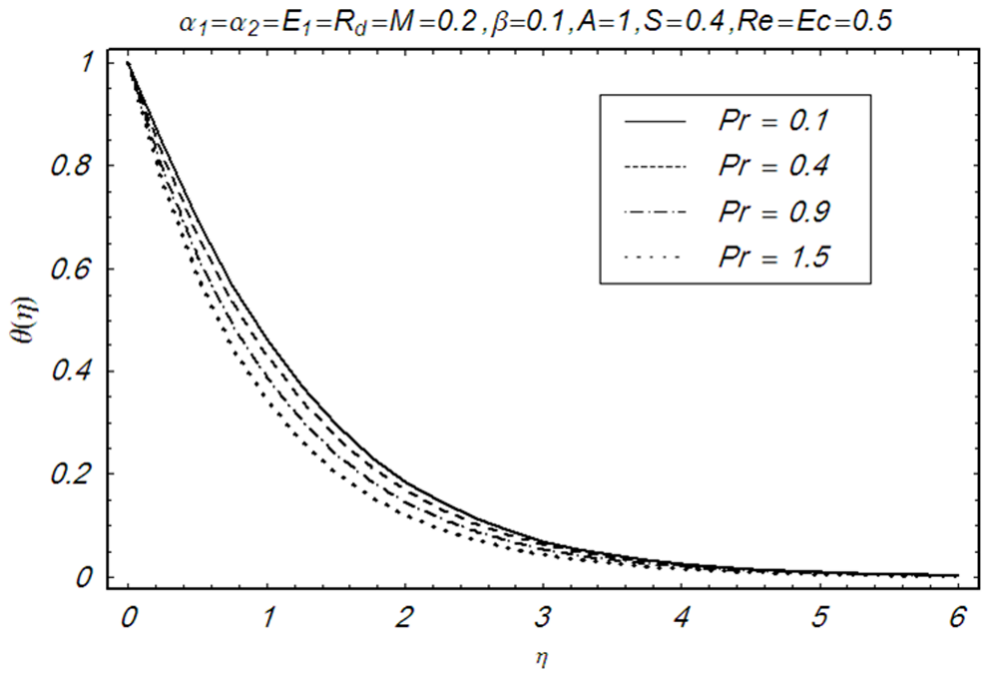
Influence of Pr on θ(η).

**Figure 20:**
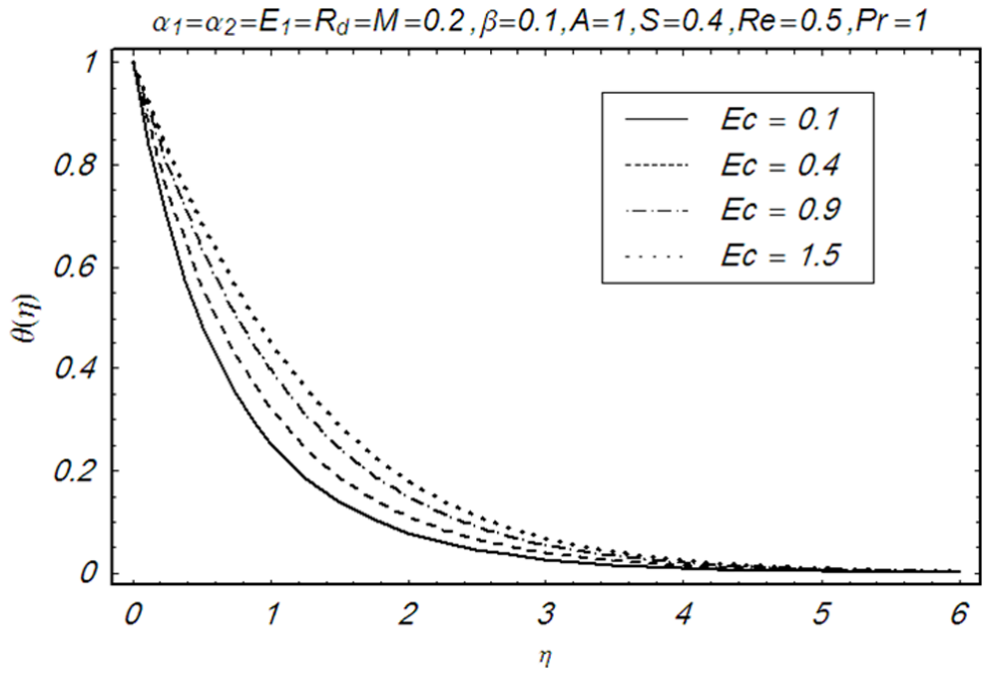
Influence of Ec on θ(η).

**Figure 21:**
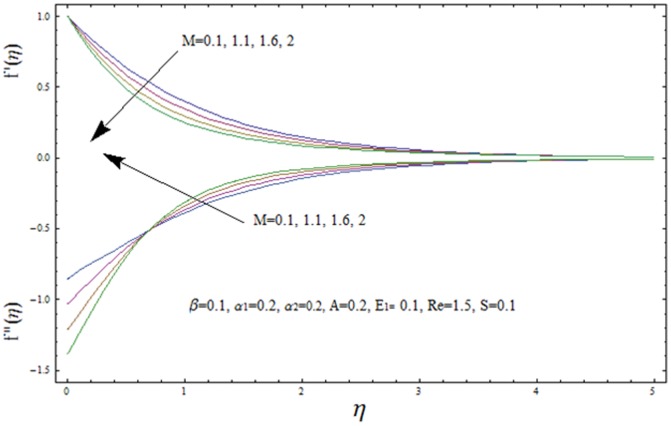
Variation of velocity f′(η) and shear stress f″(η) with η for several values of Hartman number M.

**Figure 22:**
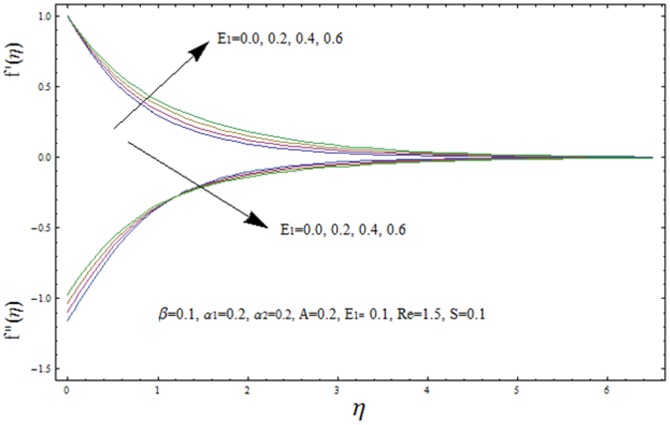
Variation of velocity f′(η) and shear stress f″(η) with η for several values of electric parameter E_1_.

This section illustrates the impact of physical parameters. The results are displayed graphically in the Figs. 2–20. The conclusions for flow field and other physical quantities of interest are drawn. The numerical values of the skin friction coefficient and local Nusselt number are presented in the Tables 2 and 3 for various values of 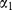, 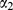, 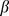, 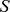, 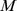, 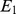, 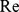, 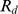, 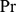 and 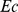. Fig. 2 displays the effect of Hartman number 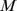 on velocity profile by keeping other physical parameter fixed. It is of interest to note that the velocity profile decreases with an increase in 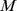 whereas the boundary layer thickness reduces. Clearly by increasing magnetic force, the Lorentz force increases which cause resistance in the fluid flow and consequently the velocity profile decreases. Fig. 3 shows the influence of third grade parameter 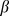 on the velocity profile 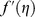. Here we noticed that the velocity increases near the wall with an increased 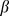 whereas it vanishes away from the wall. Figs. 4 and 5 illustrate the variation of second grade parameters 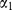 and 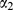 on the velocity profile 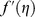 respectively. It is observed that the velocity profile 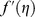 is an increasing function of 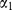. The velocity profile also increases when 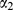 is increased. Fig. 6 is plotted for the effects of the suction parameter 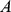 on the velocity profile 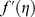. The velocity profile decreases by increasing parameter 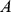 and further the boundary layer is also decreasing function of 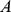. Fig. 7 is sketched for the influence of unsteadiness parameter 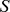 on the velocity profile. The velocity profile and the thermal boundary layer decreases for larger values of 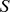. The behavior of Reynolds number 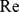 on velocity profile is shown in Fig. 8. It is observed that the velocity profile decreases with an increase in Reynold number 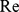. The influence of electric parameter 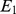 is shown in Fig. 9. This Fig explains that as the electric parameter 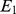 increases, the velocity boundary layer increases near the plate with small rate but increases away from the stretching plate more rapidly. In fact the Lorentz force (arising due to the electric field acts like an accelerating force) reduces the frictional resistance which causes to shift the stream line away from the stretching sheet. Fig. 10 portrays the effects of magnetic parameter 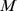 on the temperature profile 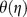. It is depicted that temperature profile and thermal boundary layer thickness increase with an increase in magnetic parameter. Fig. 11 is the plot of temperature profile 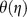 for various values of third grade parameter 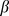. The effect of third grade parameter 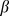 on 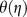 shows a decrease near the wall. The boundary layer thickness also decreases. Figs. 12 and 13 describe the effects of second grade parameters 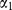 and 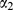 on temperature profile 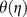. Fig. 12. depicts that the effect of second grade parameter 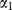 is to reduce the temperature distribution in the boundary layer which results in thinning of the boundary layer thickness. Same behavior is shown in Fig. 13 for various values of 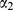. The influence of suction parameter 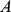 and unsteadiness parameter 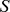 are analyzed in the Figs. 14 and 15. Here the temperature profile decreases with the increase of unsteadiness parameter 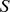 and the suction parameter 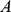. Further the thermal boundary layer also decreases by increasing both the unsteadiness parameter 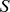 and the suction parameter 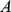. Fig. 16 shows that the temperature profile and thermal boundary layer is decreasing function of Reynold number 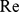. The effects of thermal radiation parameter 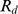 on temperature is shown in Fig. 17. It is revealed that the radiation parameter 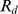 causes increase in the fluid temperature 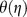. On the other hand the thermal boundary layer thickness also increases. In Fig. 18 the influence of electric parameter 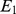 on temperature profile is given. This Fig. depicts that the temperature profile and the boundary layer thickness increase with an increase of electric parameter 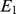. Fig. 19. illustrates the effects of Prandtl number 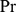 on the temperature profile 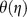. Both the temperature and thermal boundary layer thickness are decreased by increasing 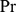. We displayed the temperature field for various values of Eckert number 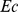 in Fig. 20. The effect of Eckert number is to increase the temperature boundary layer thickness due to the frictional heating. Fig. 21 shows the effects of Hartman number 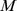 on velocity 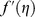 and shear stress 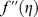. With the increase in 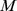, the velocity field 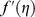 decreases near the wall and vanishes far away from the wall while shear stress 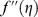 has same behavior for larger values of Hartman number 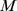. An opposite behavior is noted when 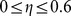. Fig. 22 demonstrates the effects of electric parameter 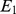 on velocity 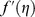 and shear stress 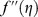. It is worthmentioning to point out that velocity is increasing function of electric parameter 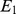 near the wall whereas opposite behavior for shear stress is observed for 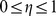. The numerical values of skin friction coefficient for various physical parameters are shown in Table 2. Here the magnitude of skin friction coefficient increases with the increase of second grade parameters (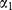, 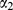), third grade parameter 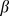, unsteadiness parameter 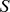, Hartman number 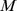 and Reynold number 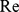 whereas it decreases with an increase in electric parameter 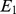. Table 3 shows the effect of physical parameters on heat transfer characteristics at the wall 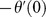. From this table, we observe that for large values of second grade parameters (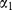, 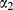), third grade parameter 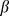, unsteadiness parameter 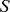, radiation parameter 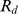 and Prandtl number 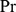 the heat transfer coefficient at the wall 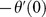 increases while it decreases for Hartman number 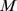, Reynold number 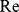, electric parameter *E_1_* and Eckert number 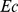.

**Table 2:** Numerical values of skin friction coefficients 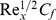 for different values of physical parameters.

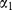	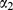	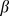	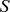	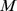	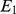	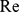	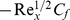
0.00	0.1	0.2	0.5	0.1	0.3	0.7	1.453
0.10							1.532
0.14							1.567
0.1	0.0	0.2	0.5	0.1	0.3	0.7	1.600
	0.1						1.632
	0.2						1.668
0.1	0.1	0.0	0.5	0.1	0.3	0.7	1.433
		0.1					1.489
		0.2					1.532
0.1	0.1	0.2	0.5	0.1	0.3	0.7	1.532
			0.6				1.592
			0.7				1.670
0.1	0.1	0.2	0.5	0.1	0.3	0.7	1.532
				0.2			1.536
				0.3			1.545
0.01	0.01	0.2	0.5	0.1	0.5	0.7	1.492
					0.6		1.487
					0.7		1.482
0.1	0.1	0.2	0.5	0.1	0.3	0.7	1.532
						0.8	1.542
						0.9	1.551

**Table 3:** Numerical values of Nusselt number 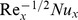 for different values of physical parameters.

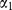	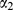	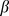	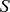	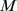	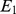	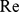	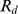	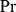	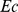	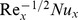
0.0	0.2	0.2	0.5	0.1	0.3	0.7	0.3	1.0	0.5	1.668
0.1										1.689
0.2										1.706
0.1	0.0	0.2	0.5	0.1	0.3	0.7	0.3	1.0	0.5	1.660
	0.1									1.674
	0.2									1.689
0.1	0.2	0.0	0.5	0.1	0.3	0.7	0.3	1.0	0.5	1.683
		0.3								1.691
		0.4								1.731
0.1	0.2	0.2	0.5	0.1	0.3	0.7	0.3	1.0	0.5	1.689
			0.6							1.805
			0.7							1.920
0.1	0.2	0.2	0.5	0.1	0.3	0.7	0.3	1.0	0.5	1.689
				0.5						1.669
				0.8						1.638
0.1	0.2	0.2	0.5	0.5	1.0	0.7	0.3	1.0	0.5	1.938
					1.5					1.889
					2.0					1.780
0.1	0.2	0.2	0.5	0.1	0.3	0.7	0.3	1.0	0.5	1.689
						1.0				1.668
						1.5				1.652
0.1	0.2	0.2	0.7	0.1	0.5	0.5	0.3	1.0	0.5	1.920
							0.4			1.991
							0.5			2.060
0.1	0.2	0.2	0.7	0.1	0.5	0.5	0.4	1.0	0.5	1.991
								1.1		2.109
								1.2		2.223
0.1	0.2	0.2	0.7	0.1	0.5	0.5	0.4	1.0	0.5	1.991
									0.6	1.938
									0.7	1.886

### Concluding remarks

The flow of third grade fluid and heat transfer in the presence of thermal radiation and Ohmic dissipation are examined. The graphs are prepared to study the influence of the pertinent flow parameters including the second grade parameter (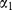, 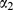), third grade parameter 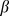, unsteadiness parameter 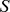, magnetic parameter 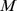, electric field parameter 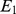, Reynolds number 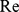, radiation parameter 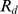, Prandtl number 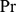 and Eckert number 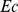. The following observations hold:

- The effect of third grade parameter 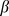 is to increase the boundary layer thickness.
- The maximum velocity is attained for higher values of electric parameter 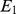.
- Effect of suction parameter, unsteadiness parameter and Reynolds number on boundary layer thickness is similar in a qualitative sense.
- Effects of 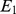 and 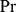 on temperature profile are quite opposite.
- The velocity field 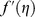 is decreasing function of Hartman number 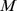.
- Magnitude of skin friction coefficient 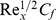 is increasing function of 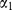, 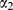, 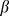, 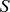, 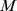 and 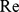.
- Electric parameter 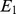 decreases the magnitude of skin friction coefficient.
